# Social stress and affiliative touch: A tale of two affective states

**DOI:** 10.1016/j.isci.2026.114833

**Published:** 2026-02-02

**Authors:** Lito Parapera Papantoniou, Orysia Vityk, Stamatina Tzanoulinou

**Affiliations:** 1 Department of Biomedical Sciences (DSB), Faculty of Biology and Medicine (FBM), University of Lausanne, Lausanne, Switzerland

**Keywords:** Neuroscience, Social sciences

## Abstract

Social interactions are an integral part in the life of social species. Humans and other species, such as rats and mice, thrive on positive social interactions, whereas stressful social encounters can be among the most negative and traumatizing experiences. These traumatic experiences have often been linked to psychopathology, with some individuals being more vulnerable than others in developing maladaptations. Here, we review literature regarding both positive and negative affective states linked with prosocial/affiliative and stressful social experiences, respectively. Of all positive social interactions, social touch is a particularly potent and evolutionarily conserved behavior associated with social buffering. We examine these topics from the standpoint of both human studies and fundamental research involving rodents, as rodents are among the most commonly used model organisms. As we explore the physiological mechanisms underlying social stress and affiliative touch, our review highlights that many common brain regions are engaged in both species examined. Moreover, a substantial overlap exists in the neural substrates involved during both positive and negative social interactions. This evidence denotes the need to refine our experimental approaches to further delineate the involvement of these areas in a cell- and projection-specific manner for positive and negative social interactions. We conclude that despite the well-known buffering effects of social touch for stress and anxiety, more interdisciplinary research is needed to establish somatosensorial approaches, such as touch-based interventions, as a standard avenue in the treatment of stress-related symptomatology. We argue that social/affiliative touch could, in fact, be one of the most effective “antidotes” in the aftermath of social stress.

## Graphical abstract

**Figure:**
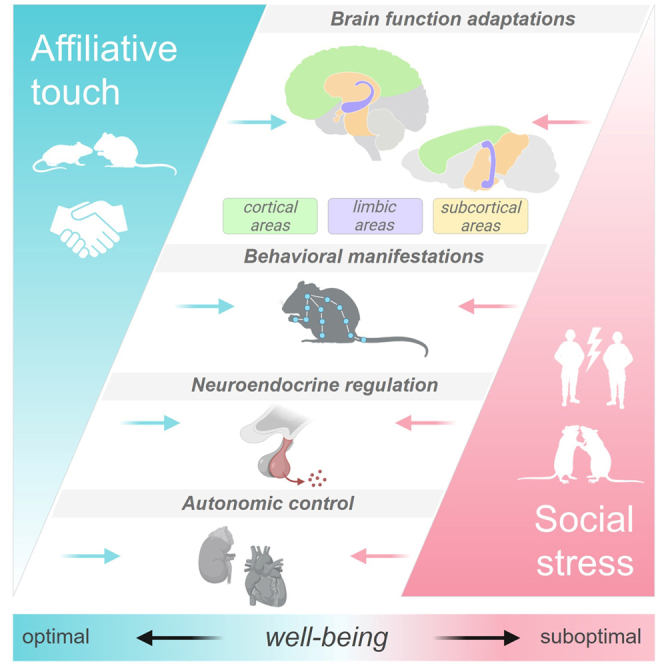

## Introduction: The diverse nature of social interactions

This review is dedicated to the memory of Maria Isabel (Maribel) Cordero for her important contribution to the stress field and her continuous enthusiasm and encouragement for scientific discoveries, big or small.

Throughout millions of years of evolution on Earth, animals have faced numerous challenges, prompting the emergence of new traits and a diverse range of defensive behaviors that have improved their species’ chances of survival.^1^ One such advantageous adaptation was the transition from a solitary lifestyle to group-living with other members of the species (or conspecifics). Research on social mammals shows that strong social ties often correlate with higher survival and reproductive rates, supposedly because of collective alertness, improved ability to defend the group from predators and optimal duty distribution allowing more efficient resource foraging and offspring care.^2^ Thus, this organization into social groups has led to the formation of bonds between members of the group and it is associated with increased adaptive value.^3^ In fact, the quality and properties of social interactions can determine critical well-being factors such as longevity and aging dynamics.^4^^,^^5^ Although significant evidence and meta-analyses have shown links between social relationships and mortality, the quality of social interactions is still not consistently recognized among the established risk factors for mortality.^6^

Nevertheless, living in social groups inevitably leads to competition for resources and mates within the group. This intra-group rivalry can give rise to aggression between individuals and promote the formation of dominance hierarchies in many animal societies, where intimidation and fighting are often used to secure status and access to resources.^7^ Thus, on the flip side of beneficial and pleasant social interactions, social stress (for relevant terminology in this review please see Box 1) is considered as one of the most widespread and intense stressors that social species can experience. It can take many forms such as war, bullying, social discrimination, and intimate partner violence and it has been often linked with anxiety- and stress-related psychiatric diseases.^8^^,^^9^^,^^10^ The concept of social traumatizing experience has been developed at the intersection of social science and psychiatry.^11^ The interplay of established societal beliefs, post-hoc processing (evaluating the danger and memory formation) and inherent factors (such as genetic susceptibility) may later result in psychopathology in a subset of individuals. Although the connection between social stress and the development of psychopathology is well-established, the exact physiological mechanisms underlying this process are still not fully understood. As a result, even though clinicians have access to various pharmacological treatments, these options are often limited in their effectiveness and are frequently associated with important side effects.

Box 1 **Terminology table**

**Table:** 

Term	Definition	References
Affective	This term is used to denote internal states such as emotions and motivation. **Positive affect** specifically refers to the experience and expression of positive emotions like joy, happiness, excitement, and enthusiasm. It’s a distinct aspect of an individual’s emotional state and is often associated with well-being and positive experiences. The term is also used for rodents.	Barrett and Bliss-Moreau^290^; Panksepp and Lahvis^291^
Affiliative	The term **affiliative** broadly relates to forming social and emotional connections or relationships with others, emphasizing social cohesion and bonding. In psychology, it specifically refers to behaviors or feelings aimed at creating or maintaining these social bonds.	Depue and Morrone-Strupinsky^292^; Wu et al.^160^
Stress/Trauma	The definition of **stress** has been constantly changing with most definitions describing it as the “fight or flight” response upon facing a threat or an event threatening for our homeostasis or more generally, as an adaptive response to an environmental stimulus. In specific cases, when stressors are intense, chronic or unpredictable, they can exceed an organism’s capacity to adapt, leading to maladaptations. In this review we use the term **social stress** to refer to stressful experiences of social nature, such as interpersonal trauma, bullying, conflict etc.	McEwen and Akil^277^; Richter-Levin and Sandi^293^
Social reward	Social Interactions or experiences that individuals actively pursue can be broadly considered as **socially rewarding**. However, there is no general consensus for a concrete definition of social reward in humans and animals.	Stijovic et al.^18^; Bhanji and Delgado^187^
Social buffering	**Social buffering** describes situations where aversive or stressful experiences are “buffered”, i.e., the stress response in an individual is reduced by the presence of another conspecific.	Kikusui et al.^294^; Gachomba et al.^149^
Valence	**Valence** refers to how a stimulus is perceived by the individual, i.e., its positive or negative quality.	Kahnt et al.^295^; Vieitas-Gaspar et al.^296^
Salience	A **salient stimulus** or event is one that stands out compared to its surroundings and captures the attention of the individuals. In other words, salience denotes the absolute intensity/importance of a stimulus, either positive or negative.	Kahnt et al.^295^; Tsai et al.^297^; Schultz^298^
Reward prediction error (RPE)	A **reward prediction error** represents the difference between the expected reward and the actual reward received. It provides a signal that updates future expectations and guides behavior, with positive RPEs happening when the outcome is better than expected and negative RPEs when the outcome is worse than expected.	Schultz et al.^188^
Discriminative touch	With the term **discriminative touch** the field refers to the kind of sensation that detects touch’s physical properties (location, shape, texture etc.)	McGlone et al.^171^; Abraira et al.^168^
Affective touch & Affectionate touch	**Affective touch** is defined as a sensation that carries emotional content, observed in a social context. **Affectionate touch** carries an intention from the provider to express love, care and appreciation among others. Representative behaviors of affectionate touch include kissing, hugging, non-sexual stroking of the back etc.	McGlone et al.^171^; Abraira et al.^168^; Jakubiak and Feeney et al.^228^; Floyd^299^
Allogrooming	**Allogrooming** or **social grooming** are terms used to denote non-aggressive physical tactile behaviors linked with hygienic purposes, but also with social bonding and reinforcing social relationships in a group.	Suvilehto et al.^153^; Morrison et al.^154^

Basic term definitions with corresponding references. The aim of this table is not to be exhaustive of the use for each concept but rather to assist the reader in navigating this review more effectively.

While social stress is well-defined in humans, a similar definition is relatively obscure in other animals due to the inability to evaluate self- and group-awareness in non-human species. However, specific behaviors have been observed in animals that seem to be caused by violence deriving from conspecifics. For example, females of different species, such as dolphins and elks, tend to withdraw from the group and migrate to less crowded areas or form separate female groups to avoid unwanted mating attempts from males.^12^^,^^13^ When studying emotions and affective states, direct access or readouts of subjective emotional experiences of other animals is restricted, as animals lack the capacity for verbal self-report.^14^^,^^15^ However, there is convincing evidence that core emotional states and their underlying neural mechanisms are evolutionarily conserved across species.^16^ Thus, approaches that bridge data from rodents and humans have the potential to yield meaningful insights and advance translational research (for a recent example see in the study by Contestabile et al.^17^). Examining social stress and social touch in a cross-species manner is going to enrich our understanding with the overarching aim to pursue impactful translational applications.

**Figure 1:**
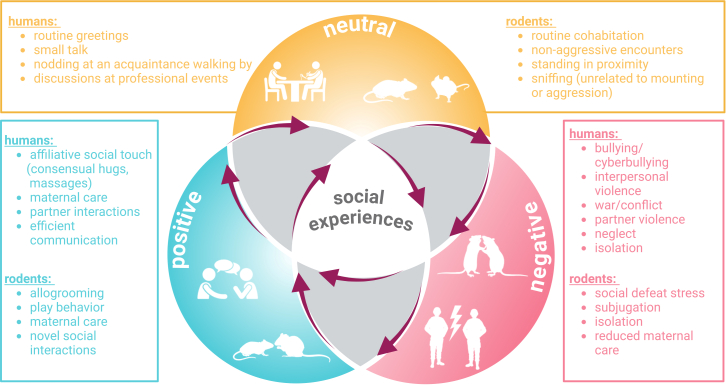
Schematic of the diverse nature of social experiences Social experiences as a continuum; examples are provided for neutral, positive and negative social interactions in humans and rodents.

Social experience can be conceptualized as a continuum, including positive, negative, and neutral social behaviors or interactions (Figure 1). Here, we aim to focus on the two extreme sides of this continuum, discussing the affiliative or rewarding social behaviors on one hand, and social stress on the other, triggering positive and negative affective states, respectively (Box 1). It should be noted that social reward is a multi-dimensional construct and no clear consensus exists for its definition, leading to great variability in study design, result interpretation and translational conclusions from rodents to humans and vice versa.^18^ For the purpose of this review, we adopt a general definition for social rewards to denote positive social experiences that individuals tend to pursue. Among socially rewarding experiences, we will zoom in on affiliative touch as a potential social buffering mechanism (Box 1). It should be acknowledged, however, that affiliative touch is not always experienced as rewarding, and in fact, in the case of autism, social stimuli including touch, may be perceived as aversive, rather than pleasurable.^19^^,^^20^ Accordingly, we will draw upon some examples from research with autism spectrum disorder (ASD) models to illustrate variations in the perception of social rewards and differences in the underlying brain circuitry.

**Figure 2:**
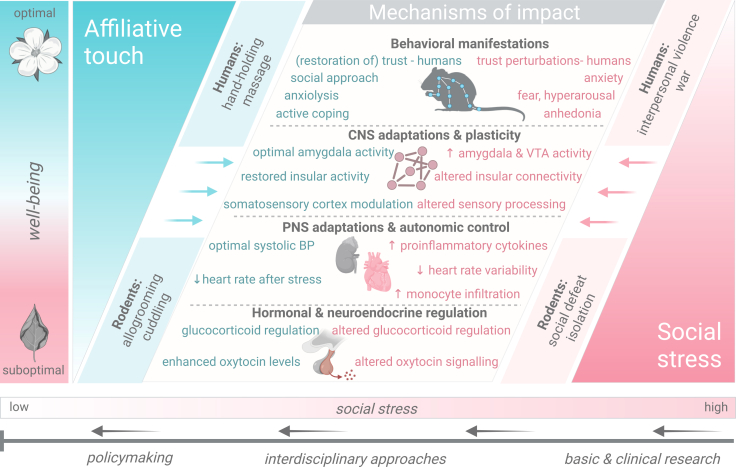
Overview of different levels of influence between positive and negative affective states pertaining to affiliative touch (left; color-coded in blue) and social stress (right: color-coded in pink) respectively According to our perspective deriving from this review, basic research and interdisciplinary approaches need to guide future policymaking by integrating programs promoting positive social interactions, such as touch-mediated approaches, as an “antidote” in the aftermath of social stress. Abbreviations: CNS; central nervous system, VTA; ventral tegmental area, PNS; peripheral nervous system, BP; blood pressure.

We note that there are several types of negative social experience, including the absence of social interactions altogether, such as, for example, in situations of social isolation/loneliness or neglect during development. The importance of social isolation or loneliness and the hypothesis of social homeostasis have been expertly reviewed previously.^21^ Rather, in this review, our focus is on social stress as an omnipresent stressful agent in society. From this perspective, social stress can be viewed as a factor that distorts the trust and positive emotions that arise from social interactions. This could imply that one efficient way to decrease the negative effects of social stress would potentially lie in actively and purposefully enhancing affiliative interactions within the social domain, reclaiming the former standing of positive interactions and promoting well-being (Figure 2). This approach can be rather challenging, however. Evidence shows that stress exposure can alter key processes and brain circuitry in ways that reduce and compromise the buffering effects of affiliative social interactions, such as touch, leading to a vicious cycle of dysfunctional processing.^22^ These topics will be briefly addressed in the following sections.

Here, we review both extremities of social behaviors leading to positive and negative affective states, what their consequences are for human and animal health, as well as the accompanying neurobiological adaptations that are known to be involved in one and the other. Finally, we discuss open questions that could be addressed from an interdisciplinary perspective and discuss how searching for these answers could help in restoring the beneficial influence of positive social interactions.

## Social stress

Stressors are an inherent aspect of life that have driven adaptations to constantly changing environments, although some stimuli or conditions can at times place demands that exceed an organism’s capacity to adapt (Box 1).^18^ According to the diagnostic and statistical manual of mental disorders (DSM-V), a traumatic experience can be seen as “exposure to actual or threatened death, serious injury, or sexual violence” experienced directly or indirectly by witnessing others.^23^ For the purpose of this review, we will use the term social stress to denote strong and intense stressors present in modern societies in multiple contexts, such as war, bullying, racism, migration, interpersonal or intimate partner violence.^10^^,^^24^^,^^25^ Notably, the dramatic increase of social-related stressors during the Sars-CoV-2 pandemic led to elevated reports of depression and anxiety symptoms,^26^ with social isolation accounting for mental health issues and increasing cases of abuse and violence due to home restriction.^27^ Exposure to stress can indeed lead to a wide range of symptoms, such as elevated anxiety, fear, withdrawal from social activities, anhedonia and hypervigilance, and potentially contribute to the development of trauma and stressor-related disorders, which include reactive attachment, disinhibited social engagement and acute stress disorders, as well as post-traumatic stress disorder (PTSD).^23^

According to the World Health Organization (WHO), 70% of people will experience a traumatic event at some point in their lives,^28^ yet, only 5.6% of them will develop PTSD.^29^ Evidence from both fundamental and clinical research reveals a combination of factors that influence vulnerability to social stress. These can include genetic and epigenetic factors, specific molecular and cellular adaptations, distinct brain plasticity mechanisms and circuitries, environmental factors, developmental trajectories, and behavioral traits associated with heightened susceptibility. Vulnerability factors of individual variability in humans and rodents are mentioned in dedicated subchapters below.

It is important to note that stress and social stress in particular, shares many overlapping mechanisms with physical pain in terms of brain regions and physiological adaptations.^30^ While acute social stressors in both clinical and preclinical settings have been linked with stress-induced analgesia, mediated mainly by the endogenous opioid and cannabinoid systems,^31^ chronic social stress can promote pain sensitization through emotional distress, stress hormone release and inflammatory changes.^32^ Pain on the other hand, can increase vulnerability to stress and disrupt social behavior, leading to conflict and social isolation, thus supporting a vicious cycle where one factor reinforces the other (for comprehensive reviews see in the studies by Sturgeon et al., Abdallah et al., and Timmers et al.^30^^,^^33^^,^^34^). Nevertheless, social support and active coping mechanisms emerge as beneficial for improving pain, and especially chronic pain perception.^30^ Despite the overlapping mechanisms between pain, physical and social stress, our focus here is social stress as it is one of the most common stressors in social species,^9^^,^^35^^,^^36^ affecting socioemotional experiences in everyday life and strongly associated with stress-related disorders.^37^^,^^38^

In rodents, social stress can be modeled with several behavioral paradigms, each of which simulates different aspects of social interactions and offers different plasticity windows for intervention.^39^ Such aspects can be early life neglect, social isolation, and aggression (for an overview of how social stress is modeled in rodents, please see Box 2).^39^^,^^40^ Aggression-based protocols are widely used to study multiple psychiatric disorders^41^^,^^42^ and seminal work on rats and mice has established social defeat as one of the most recognized models for social stress in rodents.^43^^,^^44^^,^^45^^,^^46^

Box 2 **Overview of social stress paradigms**

**Table:** 

Paradigm	References
**Chronic Social Defeat Stress (CSDS):** The experimental mouse is introduced in the home cage of an aggressive mouse (usually a CD1 retired breeder) and becomes the target of several attacks. The protocol typically lasts for 10 min per day for a total duration of 10 days. It is well-described to promote social avoidance, depressive-like and anxiety-like behaviors, as well as physiological alterations in the central and peripheral nervous systems.	Berton et al.^45^; Golden et al.^46^; Krishnan et al.^93^; Kudryavtseva et al.^300^
**Subchronic social defeat stress:** The procedure is similar as the one described in the CSDS above, but with reduced duration and/or intensity of the stressors, e.g., 5 instead of 10 days, thus minimizing the probability of excessive wounding. In the **microdefeat** protocol in particular, the experimental mouse receives three 5-min long social defeat sessions on the same day. Social interaction is tested the following day. This procedure is considered as subthreshold and is not reported to result in social avoidance.	Krishnan et al.^93^; Goto et al.^301^; Otabi et al.^302^; Goto and Toyoda^303^; Larrieu et al.^304^; Christoffel et al.^305^
**CSDS variations:** Multiple variations of the CSDS exist. For example, in the **vicarious social defeat stress**, the experimental mouse receives emotional stress by witnessing the defeat of another conspecific, resulting in social avoidance, depression and anxiety-like behavior.	Sial et al.^306^; Takahashi et al.^133^
**CSDS variations in females:** Eliciting aggression toward female rodents is proving more challenging, therefore several modifications have been applied to the CSDS toward this aim. In the **non-discriminatory social defeat stress** a pair of a male and a female mouse are simultaneously introduced into the home cage of the aggressor. The protocol leads to reduced sociability, depression and anxiety-like behavior in both sexes. Other adaptations include application of male odorants to females and lead to social avoidance, depression and anxiety-like behavior. Optogenetic activation of a specific cell population in the aggressor’s VMHvl (region important for aggression) has also been proven efficient in eliciting aggression toward female subjects.	Yohn et al.^307^; Harris et al.^134^; Lee et al.^308^
**Early Life neglect: Maternal separation** includes separating the litter from the dam for at least 3 h per day during the first 2 weeks post-birth. It leads to diminished social interactions later in life. In the **early social deprivation** protocol, the pups are separated from both the dam and their littermates during the first 2 weeks post-birth and the protocol results in altered social behavior and increased aggressiveness in adulthood.	Sandi and Haller^39^; Levine^309^; Ader^310^; Hofer^311^
**Post-weaning social isolation:** The experimental subject undergoes isolation rearing starting from the weaning period (around P23) until early adulthood. This results in increased sociability and aggression later in life.	Sandi and Haller^39^; Valzelli^312^

Brief description of the main paradigms used to model social stress in rodents with corresponding references.

Animal models have deepened our understanding regarding crucial physiological mechanisms affected by social stress and have contributed to major clinical advancements. Current treatment options for trauma- and stressor-related disorders such as PTSD include a combination of behavioral treatments, mainly cognitive behavioral therapy (CBT), eye movement desensitization and reprocessing (EMDR), with pharmacological agents, such as MDMA-assisted therapy, antidepressants, glucocorticoids, acute ketamine administration, or short-term use of anxiolytics.^47^^,^^48^^,^^49^^,^^50^^,^^51^ However, several of these treatments, such as the selective serotonin reuptake inhibitors (SSRIs), a class of antidepressants administered as a first line medication for PTSD, are proving to be ineffective in the long-run for many patients.^52^ Given the increased individual variability in response rates and the diverse nature of its sources, the search for neurobiological mechanisms of stress-related disorders remains pertinent for fundamental and clinical research alike. Further we review briefly how brain areas and physiology are affected by social stress in humans and rodents. Regarding humans, many of our references focus on PTSD-related findings, as it is a prevalent stress-related disorder with extensive post-diagnosis reports and evidence.

## Physiological and neurobiological alterations upon social stress exposure

### Periphery-related alterations linked with social stress in humans

Fundamental work since the 1980s has revealed that intense stressful experiences can alter the brain and body by disrupting key brain circuits and stress-responsive structures, potentially leaving a long-lasting mark that can underlie pathological responses, like those observed in PTSD.^53^ Indicative of these systemic responses after psychosocial stress, there are documented changes in heart rate variability, as well as increased levels of pro-inflammatory molecules in the blood, such as interleukin 6 (IL-6), tumor necrosis factor α (TNFα) and interleukin 1β (IL-1β).^54^^,^^55^ These molecules can then cross the blood-brain barrier (BBB) to interfere with systems affected by disease, as in the case of major depressive disorder (MDD).^55^ In this context, neuroinflammation becomes evident by elevated pro-inflammatory cytokine levels, microglia activation, and peripherally-derived monocytes and macrophages invading the brain.^56^ Increased IL-6, IL-1β, TNFα, and interferon γ levels have been identified in patients with PTSD, with this low-grade inflammation profile being considered as a potential biomarker for susceptibility to the disease.^57^ Moreover, systems-level multi-omics profiling of active-duty participants with recent PTSD and veterans with chronic PTSD conditions revealed that the latter had, among others, increased chronic inflammation signatures and oxidative stress compared to the former.^58^ These alterations were associated with comorbidities and possibly, with the progression and severity of the disease.^58^ Pioneering studies on Holocaust survivors and their offsprings revealed a subset of immune- and glucocorticoid-related genes to be associated with intergenerational trauma.^59^ Lastly, a recent brain-omics approach including brain tissue and blood from patients with MDD or PTSD resulted in an updated atlas with integrated multi-level information and of note, the dentate gyrus of PTSD patients emerged as a methylation hotspot.^60^

### Brain circuitry relevant to social stress: Evidence from human studies and disease

Exposure to stressful life experiences (especially of social nature in the case of humans) serves as a common precipitating event across mood, fear, and stressor-related disorders.^61^^,^^62^^,^^63^^,^^64^ Apart from convergent triggering pathways, the symptomatology of the previous diseases also presents significant overlap.^23^^,^^65^^,^^66^^,^^67^^,^^68^ Therefore, it may be further hypothesized that the biological substrates of such pathological manifestations could share some degree of similarity. In fact, a recent multi-omics analysis of both blood and brain samples from patients with depression or PTSD revealed both unique and shared molecular signatures between the disorders.^60^ Nevertheless, individuals exert high heterogeneity in social stress responses, with some patients exhibiting for instance MDD, while others being diagnosed with PTSD upon the same traumatic event and other people not meeting the criteria for neither of these disorders.^69^ The source of this individual variability constitutes an intricate topic and is discussed in a dedicated chapter further. Rather than concentrating on one particular disorder, we seek to present evidence from circuits affected across several of these conditions, triggered by socially stressful events, with the aim to present an overview of the brain areas/circuits affected by social stress.

Neuroimaging studies in humans provide elaborate insights into brain circuitries affected by social stress. Amygdala constitutes the major fear processing hub of the brain, receiving cognitive control from the prefrontal cortex (PFC).^70^ Higher amygdala activation, revealed via elevated regional cerebral blood flow (rCBF), along with reduced medial PFC (mPFC) activity, has long been demonstrated in both male Vietnam combat veterans and female nurse veterans with PTSD.^71^ Social stress in the form of social rejection, social evaluation, and racism-related stress, has been documented to engage areas like the anterior cingulate cortex (ACC), PFC (especially the ventrolateral PFC or VLPFC), bilateral insula, and thalamus, and also alter their functional connectivity.^72^ A seminal meta-analysis demonstrated increased insula activation in patients with PTSD, social anxiety disorder and specific phobia,^73^ and later findings revealed heightened insula connectivity with basolateral amygdala and other subcortical structures, such as periaqueductal gray (PAG), potentially contributing to hypervigilance, emotional distress, and aberrant emotional processing.^74^^,^^75^ Moreover, functional near-infrared spectroscopy (fNIRS) during the Trier social stress test (TSST), a task widely used to elicit uncontrollable social-evaluative threat in humans, demonstrated increased activation of the dorsolateral prefrontal cortex (DLPFC), superior parietal cortex, and inferior frontal gyrus compared to control conditions, areas which constitute parts of the cognitive control and dorsal attention networks, and suggest adaptive changes under an emotionally and cognitively challenging environment.^76^ In addition, it was recently reported that connectivity reorganization of resting-state brain networks, namely the salience (SN), central executive (CEN), and default mode networks (DMN), upon acute psychophysiological stress during a socially evaluated cold pressure task (SECPT), is dependent on cortisol responsiveness.^77^ Social stress was also shown to result in connectivity reorganization between the left DLPFC and the arousal network, as well as the right inferior temporal gyrus (ITG) and the DMN, with these changes being associated with future PTSD symptom severity and depressive symptoms.^78^ Upon acute stressful psychological and emotional experiences, recapitulated by viewing aversive movie clips representing social stress scenarios (“extreme male-to-male aggressive behavior and violence in front of a crowd”), the activity of locus coeruleus (LC), which is the main source of noradrenaline in the brain, has been shown to increase.^79^^,^^80^ This heightened noradrenergic activity alters the functional connectivity of brain areas, such as the hypothalamus and midbrain, that are involved in attentional shifting, vigilance perception, and autonomic-neuroendocrine control.^80^ It is important to note that viewing of disturbing audiovisual material, even in the case of depicting scenes of social stress, is not the same as experiencing social stress per se, e.g., the former lacks the pain/sensory processing part, however, it is a commonly used approach in human research that can provide information regarding partially overlapping activated modalities. Studies also reveal that PTSD patients show alterations when processing stimuli for their valence. For instance, a functional magnetic resonance imaging (fMRI) study, performed when individuals experienced stimuli relevant to social evaluative threat, revealed reduced hippocampal activity, indicative of perturbations in processing negative feedback.^81^ In relation to reward deficiencies in the aftermath of social stress, another fMRI study on social stress victims that had been exposed to interpersonal assault, threatening experiences during the Bosnian war, or witnessing the death of family members, and who met the PTSD diagnostic criteria, identified reduced nucleus accumbens (NAc) and PFC activation during reward processing, suggesting underlying anhedonia/motivation deficits.^82^ Given the common occurrence of depression-related comorbidities in PTSD, there are open discussions in the field about whether the core mechanisms of reward deficits are overlapping between PTSD and MDD.^60^^,^^83^

In summary, neuroimaging studies coupled with ethological behavioral protocols have revealed important insights on activity and connectivity changes in key brain regions and networks affected by social stress that are involved in stress-evoked maladaptations, such as pathological fear, anxiety, hyperarousal, avoidance, and depressive-related symptoms. However, the differentiation between acute and chronic conditions, along with pathological comorbidities, add complexity in the effort to disentangle social stress neurobiology, especially when attempting to infer causality in the observed adaptations. Thus, further research integrating information from animal models and humans is necessary.

### Periphery-related alterations upon social defeat in rodents

Several factors render the investigation of social stress pathophysiology in humans a challenging endeavor, with the genetic background, environmental conditions, and developmental periods, among others, heavily influencing stress responses. Additionally, the range and intensity of stressful stimuli, along with the temporal constraints commonly inherent in human studies for practical reasons, often limit the amount of information that can be obtained.^47^ Thus, animal models offer important advantages to study mechanisms and can uncover interventional routes by tightly controlling the experimental conditions, developing new behavioral protocols and manipulating brain area activation/inhibition in a cell-type and region-specific manner.

Over the past decades, many preclinical and clinical studies have incorporated fear conditioning to model stress in rodents and have identified crucial nodes, such as the mPFC, amygdala, hippocampus, bed nucleus of the stria terminalis (BNST), and PAG.^84^^,^^85^ Fear conditioning is a type of associative learning whereby a neutral stimulus acquires valence through its association with a highly salient stimulus, usually a footshock.^86^ Despite the overlapping brain circuitry involved in fear conditioning protocols as described previously, and social defeat paradigms,^87^^,^^88^^,^^89^ recent evidence suggests that the brain circuitries supporting social stress and electric shocks are not fully aligned.^90^ Further we review brain areas involved in social defeat stress in rodents and we also mention recent evidence^40^^,^^90^ where circuitry involved in fear conditioning and social stress is studied and compared explicitly for the two processes.

In the present review we focus on social aggression during adulthood usually modeled in rodents with social defeat, based on the classical resident-intruder paradigm, whereby aggression is exerted by a mouse known to demonstrate higher aggression when an intruder enters its territory^46^ (please see Box 2 for more details). Acute social defeat, as other stressors, activates the hypothalamic-pituitary-adrenal (HPA) axis and sympathetic nervous system to promote physiological changes, glucocorticoid release and eventually enhance monocyte infiltration in the circulation; processes that diminish over time through a glucocorticoids-mediated negative feedback cycle.^91^^,^^92^ Chronic social defeat stress (CSDS) is known to promote generalized avoidance and depressive-like episodes that can persist for days, including anhedonia-like symptoms, reduced exploratory activity, hyperactivation of the HPA axis, decreased heart rate variability and a metabolic syndrome characterized by increased food intake and leptin resistance in more susceptible individuals.^45^^,^^46^^,^^93^^,^^94^ Of note, social defeat elicits variable responses across mice populations, with some individuals exerting marked vulnerability characterized by the aforementioned symptoms and others considered as resilient, lacking the social avoidance and anhedonia-like phenotypes, yet retaining the increased anxiety-like behavior.^69^^,^^93^ Multiple studies indicate that CSDS is associated with sensitization of central and peripheral immune cells,^95^ with diminished BBB integrity and enhanced permeability.^96^^,^^97^ Chronic social adversity also leads to macrophage infiltration, rapid proliferation, and morphological activation of microglia in the mPFC, thus regulating neuronal plasticity.^98^ Differences in stressor intensity and duration lead to differential microglia activation, with persistent activation being linked to increased pro-inflammatory markers release, enhanced phagocytic activity, increased cell-death (both neuronal and microglial) and depressive-like symptoms.^98^ CSDS has also been shown to promote oxidative stress in microglia both during and after stress exposure, often accounting for the observed behavioral phenotypes.^99^ Astrocytes constitute another important cell population implicated in social stress and depression, with the levels of the astrocytic cAMP response element-binding protein (CREB) being elevated in the NAc of susceptible individuals upon CSDS.^100^ Therefore, it becomes evident that social defeat elicits robust activation of both central—affecting both neuronal and glial cell populations—and peripheral systems, and when sustained over time, this can lead to dysregulation of these systems accompanied by profound behavioral effects. Next, we take a closer look at central nervous system alterations, reviewing brain circuitry changes in the aftermath of social defeat.

### Brain circuitry involved in models of social defeat

Delineating the brain circuits supporting responses to social defeat provides valuable information regarding the neurophysiological mechanisms of social stress that could potentially uncover neural systems/neurotransmitters relevant for interventions. Pioneering work tracing back to almost two decades ago reveals specific molecular adaptations of the mesolimbic dopamine system following CSDS. More specifically, increased brain-derived neurotrophic factor (BDNF) release to the NAc was linked to augmented social stress-induced firing of the ventral tegmental area (VTA).^93^ BDNF is a molecule important for neural growth and survival which plays crucial roles in learning and memory processes.^101^ Of note, this adaptation was observed only in susceptible mice exhibiting social avoidance, anhedonia- and anxiety-like symptoms, and not in resilient individuals who exhibited only anxiety-like symptoms. These changes were accompanied by activation of downstream molecules, and transcriptional changes in several genes implicated in depression.^93^ These findings highlight the importance of plasticity within the brain’s reward circuitry in dynamically regulating emotional states upon social challenges and paved the way for subsequent studies on stress vulnerability and resilience. In fact, the nature of this increased VTA firing has been proposed as a potential target of novel antidepressant agents, however, relevant studies have faced delays due to various regulatory and financial challenges.^69^ Regarding firing patterns and projection targets of the VTA, phasic, but not tonic, activation of VTA-to-NAc dopamine neurons (DA), or optogenetic inhibition of VTA-to-mPFC DA neurons, were both shown to induce social avoidance in mice previously exposed to subthreshold social defeat.^102^ These findings revealed novel signatures of cell-specific firing patterns associated with social stress-induced depressive-like behavior. Last but not least, DeltaFosb, a marker of long-term neuronal activation^103^ was shown to increase in the NAc of male mice upon social defeat and this increase negatively correlated with social interaction, denoting susceptibility.^104^

In addition to the mesolimbic system, other brain regions have been linked to responses to social defeat stress. A thalamocortical projection was recently implicated in the depressive-like symptomatology of CSDS.^105^ Specifically, reduced input from the mediodorsal thalamus (MDT), a higher-order thalamic relay area implicated in motivation processing, to the mPFC, resulted in an excitation-inhibition imbalance at the level of mPFC pyramidal neurons. Restoring this imbalance ameliorated the defeat-induced depressive-like phenotype, suggesting that boosting the connectivity between MDT and mPFC could be a promising target for treating depressive-like symptoms of stress-related disorders.^105^ Moreover, zona incerta (ZI)—a subthalamic, largely inhibitory region—participates in the induction of conditioned place aversion in the aftermath of CSDS and has been linked with PTSD-like symptoms.^106^ Cortical and brainstem regions are crucially implicated in social defeat-elicited responses, with reduced functional connectivity between mPFC and dorsal periaqueductal gray (dPAG) shown to promote social avoidance upon repeated social defeat.^107^

On the other hand, the hypothalamus, a central node for internal state balance, and specifically its lateral subdivision (lateral hypothalamus: LH) is crucially involved in cognitive functions and stress responses,^108^ releasing orexin peptides to control arousal, emotional regulation, feeding and adaptation to challenges.^109^ Interestingly, reduced orexinergic input from the LH to the lateral habenula (LHb), a brain region implicated in both reward and aversion, was found to be involved in anxiety and depressive-like behavior upon social defeat, with social avoidance and anxiety-like behaviors being rescued by optogenetic activation of this projection.^110^ LHb constitutes a hub linking the limbic forebrain with midbrain monoaminergic systems, often affected in mood disorders, and recent evidence demonstrates distinct LHb projections undergoing changes in synaptic transmission upon CSDS.^111^^,^^112^ More specifically, susceptible mice were found to exhibit postsynaptic long-term depression (LTD) of LHb neurons projecting to the VTA, in the context of associative learning and defensive behavior. On the other hand, this susceptible phenotype was linked to postsynaptic potentiation (LTP) in a subset of LHb neurons innervating the dorsal raphae nucleus (DRN).^112^ These data reveal novel projection-specific adaptations that impact monoamine-producing centers and that are linked with susceptibility to social defeat stress. Apart from pathway-specific alterations, cell-specificity seems to be equally important in defining alterations following social stress. The ventrolateral part of the ventromedial hypothalamus (vlVMH) was shown to encode responses to social threat and contextual cues, promoting adaptation and survival to a changing environment.^113^ Another study highlighting the importance of cell-specificity revealed long-term potentiation (LTP) upon social defeat of an oxytocinergic population in the retrochiasmatic supraoptic nucleus (SOR^OXT^) projecting to oxytocin-receptor expressing neurons in the anterior part of the ventromedial hypothalamus ventrolateral part (aVMHvl^OXTR^). These changes were not observed following footshocks or predator threat, emphasizing the unique nature of adaptations to social stress.^90^

Rodent studies are achieving important levels of detail, and an expanding body of research is beginning to systematize the neurobiological correlates of social defeat.^69^^,^^114^^,^^115^^,^^116^ That being said, we still have many unknowns regarding the cellular, molecular, and circuit mechanisms of acute and chronic social stress, as well as the degree of overlap between pathways involved in social stress and those in traditional fear processes.

## Vulnerability to social stress and inter-individual variability

As mentioned earlier, individuals vary markedly in the way they respond to social stress, with some individuals engaging in adaptive coping behaviors and others developing maladaptive responses.^69^ Factors associated with vulnerability and resilience include a complicated interplay between genetic and epigenetic factors, distinct molecular changes, plasticity/circuit adaptations, environmental influences, and developmental trajectories.^117^^,^^118^ Further we present examples of some determining factors regarding outcomes to social stress vulnerability in humans and rodents.

Biological sex constitutes a strong example whereby the genetic background differentially predisposes women to developing mental disorders, such as depression and anxiety, compared to men.^119^^,^^120^ Along the lines of genetic factors, a single-nucleotide polymorphism (SNP) in the human BDNF prodomain has been found to promote increased BDNF release to the NAc and subsequent susceptibility to chronic social stress. This increased BDNF release was also present in postmortem brain tissue of patients with depression.^93^ Regarding cellular adaptations, hippocampal neurotoxicity in response to stress has long been proposed to relate with pathophysiological changes in MDD associated with chronic glucocorticoid dysregulation.^121^ Regarding molecular underpinnings of social stress, fluctuations in peripheral inflammation levels in patients with depression, mirrored by elevated circulating IL-6, were found to positively correlate with treatment resistance.^122^ However, it is not clear whether variability in patient immune markers pre-exists and therefore can be a predisposing factor, or whether these alterations occur as a result of stress exposure. As for plasticity and circuitry adaptations, region activation and functional connectivity of several networks, including the salience network and DMN, in response to psychosocial stress were revealed to predict individual differences regarding stress vulnerability.^123^ On the other hand, exposure to stress can occur during different critical periods, e.g., during pre-conception, *in utero*, or post-conception, resulting in multiple possibilities for epigenetic changes in somatic cells or gametes that can promote transgenerational transmission of stress effects.^124^ Human studies on postmortem tissue of suicide victims with childhood trauma revealed increased methylation of the glucocorticoid receptor (GR) promoter in the hippocampus.^125^ Of note, trauma- and stress-related disorders are the only category in the DSM-V that is defined by external triggering factors.^23^ From early life adversities to living in combat zones, lacking an encouraging social cycle to seek active coping mechanisms, or being exposed to pollutants, the environment is documented to greatly impact the way someone responds to adversity.^126^^,^^127^^,^^128^ Biological age is another important factor at play and accumulating evidence indicates childhood and adolescence as developmental periods with higher probability of developing PTSD in the face of social adversities.^129^ Lastly, accumulating human and mouse evidence places highly anxious individuals at risk for developing mood disorders.^130^^,^^131^

Women are more prone to developing mental health disorders compared to men,^132^ nevertheless, basic research on aggression has predominantly included male subjects. Many of the behavioral paradigms recapitulating social stress in male rodents have not been validated in females, and those who have, often report different behavioral profiles and suggest necessary modifications to be taken into consideration for experimental design.^133^^,^^134^^,^^135^^,^^136^ The inconsistency of female data in replicating well-described male protocols of social stress may partially be due to inadequate male-centered metrics that are traditionally used to capture the qualitatively different responses of females. Indeed, it was recently reported that social stress in female mice affects the velocity rather than the actual social interaction ratio that usually serves as a social avoidance proxy.^137^ Another study in female Syrian hamsters failed to report persistent defensive or submissive behaviors upon chronic social defeat, however, demonstrated elevated adrenocorticotropin-like immunoreactivity, suggesting a dissociation between behavioral and endocrine manifestations of stress responses between sexes.^138^ Apart from sex differences, the influence of the genetic background in differentially predisposing to social avoidance and metabolic changes upon social stress is also evident when comparing different mouse lines.^139^ Moving on to the cellular level, divergent activation and expression profiles were revealed for dopamine 1 (D1) vs. dopamine 2 (D2) receptor-expressing medium spiny neurons (MSNs) in the NAc of more susceptible as compared to more resilient mice in the aftermath of social defeat, with activation of D2-MSNs being associated with susceptibility.^140^^,^^141^^,^^142^ Molecular alterations regarding the levels of key metabolites, for instance glutamine, glutamate, glycine, and choline among others, have also been reported in stress-responsive brain regions of susceptible mice in particular upon social defeat.^143^ In addition, evidence from susceptible mice elucidates inputs from neurotensin neurons of the lateral septum (LS) to the NAc upon CSDS, mediating the perception of previously rewarding stimuli as threatening and reflecting specific circuit adaptations.^144^ On the other hand, CSDS was found to alter the DNA methylation landscape in the NAc of susceptible mice, with affected genes including the estrogen receptor 1 (Esr1), known to be implicated in a range of psychiatric diseases such as depression.^145^ Regarding environmental insults, models of early life stress have been well-documented to predispose to heightened vulnerability to subsequent social defeat in adult life, highlighting the importance of early environment for normal development and healthy coping.^146^ Last but not least, another crucial trajectory involves aging, as the latter was shown to be associated with exacerbated depressive-like symptoms upon CSDS, shown to be due to neuroinflammatory processes.^147^ The ability to maintain relatively normal functioning under high stress can be at least partially learned^69^ and thus, discovering reliable biomarkers of stress vulnerability could greatly impact clinical practice.

## Positive social interactions and affiliative touch

Affiliative and prosocial interactions are critical for the well-being of most social species, and they are processed in the brain in a unique way, integrating multiplexed information from all sensory modalities.^42^^,^^148^ Humans and rodents have been shown to demonstrate prosociality in multiple contexts^149^ and, in fact, several studies have demonstrated links between the level of social integration, health outcomes and mortality rates.^6^^,^^150^^,^^151^^,^^152^ Specifically, in their seminal article, Berkman and Syme (1979) found that the less well socially-connected the individuals, the higher the mortality rate of the population, and this association persisted after corrections for physical health status, socioeconomic situation, health practices and access to health services.^150^ Other research has also substantiated these results,^151^ with a more recent meta-analysis, including 148 studies, concluding that the quality of social relationships can be a determining factor for mortality risk as other well-established risk factors, such as smoking or physical activity.^6^ However, the mechanisms via which social support seems to improve health outcomes, or asked in the inverse fashion, the mechanisms by which poor social ties lead to increased mortality, have not been delineated yet. Answering this question is critical as it will allow explicit and precise testing of the hypothesis that social buffering can indeed affect susceptibility to disease and thus aid to guide policies and treatments strategies.^150^^,^^152^

Among positive social interactions, touch is recognized as a major contributing factor to forming and maintaining attachment bonds across species.^153^ Touch can be either discriminative or affective in nature (Box 1), with each kind carrying different information and properties. In humans, social touch can take the form of gentle stroking, caressing, hugging, patting, and cuddling, and can be involved in a variety of occasions, such as comforting gestures of patting on the back between friends, or soothing parenting touch toward their offspring. In primates, mice and other species, allogrooming (or social grooming) seems to be a very important aspect of affective touch.^153^ Grooming and allogrooming behaviors in animals have been considered to serve primarily hygienic roles such as cleaning of the fur and removing parasites^154^ (Box 1). Interestingly, however, it was documented that whereas some primate species dedicate around 20% of their time to grooming and allogrooming activities daily, the amount needed for strictly hygienic purposes is far less (with time devoted to allogrooming not correlated with body mass), suggesting functions beyond hygiene.^155^^,^^156^ Rather, in social species, including nonhuman primates and mice, affective touch and allogrooming seem to play key roles in reinforcing bonds *between* individuals and *within* social groups.^153^^,^^155^^,^^157^ Thus, beyond hygienic or social bonding purposes, there seem to be additional key functions associated with affective social touch, such as mitigating psychological distress and pain that has been documented in humans,^158^ voles,^159^ and mice.^160^ Reduced engagement in allogrooming behaviors in humans^153^ complicates the ability to extrapolate the significance and relevance of this behavior from studies in rodents and nonhuman primates, highlighting the need to carefully consider the unique social context of human social touch interactions. To a certain degree, the social bonding and communicative functions carried by allogrooming in nonhuman primates and rodents have been proposed to be replaced by other behaviors, such as casual social talk, in humans.^153^ Nevertheless, despite the fact that humans are generally considered to engage less in this type of behavior, some authors emphasize the importance of social grooming in our species, providing evidence that, in fact, under certain conditions, humans engage in social grooming similarly to other primates.^155^^,^^161^ Along these lines, it has been proposed that social grooming can contribute directly to forming and maintaining trust bonds between individuals accompanied and supported by physiological and neuroendocrine cascades.^155^

On the other hand, lack of social touch can have severe negative consequences. Indeed, the detrimental effects of social isolation due to the Sars-CoV-2 pandemic are still becoming evident as the incidence of psychiatric disorders has significantly increased.^162^ In the clinic, this lack of physical touch from physicians to patients in the post-pandemic era is hypothesized to have serious adverse consequences that are not researched sufficiently yet. Further we review briefly the neurobiology of social touch and describe some of its documented beneficial effects.

In the field of behavioral neuroscience, precise measurements of social interactions and touch behaviors in rodents have posed a significant challenge, with traditional 2D-tracking methods being prone to occlusions of body parts.^163^ However, multiple efforts, both in terms of developing computer vision and machine learning approaches during freely moving social interactions, but also in developing novel tasks that assess social behaviors in a precise and controlled manner, can aid in advancing social behavior assessment, including touch-relevant repertoires.^20^^,^^163^^,^^164^^,^^165^ Similar advancements incorporating machine learning approaches have been also achieved in the field of human computational ethology and can be utilized to study sophisticated behaviors in humans.^166^ Indeed, markerless movement analysis revealed distinct biomechanical properties of hugging depending on personality traits and the relationship between individuals, revealing quantifiable aspects that would be inaccessible via traditional analysis methods.^167^

## Physiological and neurobiological mechanisms of social touch

Pleasant social touch by default, involves integration of information from multiple internal and external sources, including sensory information, internal states, and dedicated neurocircuitry.^157^ The key brain areas and circuitry involved in positive or rewarding social experiences have been the objective of several studies in humans and animals. Here, we focus on humans and rodents, firstly by giving a brief overview of peripheral physiology processes pertaining to positive social interactions and affiliative social touch and secondly, by reviewing the relevant brain areas.

Touch perception involves several steps from the periphery to the central nervous system, starting with the activation of sensory neurons and receptor activation in the skin, consequently neural transmission to the spinal cord, brainstem, and thalamus, and finally transmission and signal processing within the somatosensory cortex and the insula.^168^^,^^169^^,^^170^ Although a detailed description of the physiology of touch is outside the scope of the present review, below we provide an overview of the key components that contribute to tactile perceptions, particularly those of affiliative touch.

### Peripheral encoding of touch

Understanding social touch, and specifically affective and affiliative touch (Box 1), involves multiple levels of analyses such as perception of external cues and their integration in the experienced outcome through brain-periphery signal integration.^154^^,^^171^ Touch neurons have been recognized in humans and mice to drive signals from the periphery to the brain. In both humans and mice, specialized neurons in the skin detect gentle, pleasant touch, and relay this information through distinct neural pathways to the brain.^171^ Specifically, peripheral sensory detection via touch neurons is initially relayed to the spinal cord for signal integration which then ascends to brain areas for further processing.^153^^,^^168^^,^^172^

In humans, C-tactile afferents, an unmyelinated set of nerve fibers found in hairy skin, mediate affective touch sensations, projecting to brain regions involved in emotional and social processing.^153^^,^^172^^,^^173^ C-tactile afferents are most responsive to gentle, slow stroking (1–10 cm/s), in contrast to fast and heavily myelinated A fibers (and specifically Aβ fibers) that seem to respond to different stroking speeds, encoding mainly aspects of discriminative touch (Box 1).^153^^,^^174^^,^^175^ It should be noted, however, that A fibers seem to contribute to the perceived affective touch properties by C-tactile afferents, suggesting an important supporting role.^176^

In mice, there are cells hypothesized to be equivalent to C-tactile afferents, such as Mrgprb4-lineage neurons, located in the dorsal root ganglia (DRG) and innervating the skin.^154^ These G protein-coupled receptor (GPCR)-expressing cells are responding to gentle stroking, result in behaviors suggesting rewarding properties (e.g., gentle stroking results in conditioned place preference) and their optogenetic stimulation results in conditioned place preference and lordotic posture, suggesting increased sexual receptivity.^177^^,^^178^^,^^179^ Ablation of Mrgprb4 neurons in females using a cre-dependent diphtheria toxin resulted in reduced sexual responsivity suggesting they are required for these rewarding aspects of affective touch.^179^ Optogenetic or chemogenetic activation of Mrgprb4 neurons can reduce stress hormone levels, such as corticosterone, and promote stress resilience, demonstrating a direct skin-brain pathway for soothing touch.^180^ Another group of GPCR-expressing neurons, GPR83, seem to convey cutaneous signals from the spinal cord to the lateral parabrachial nucleus of the pons.^181^ It was shown, in fact, that Gpr83+ neurons are positioned downstream, receiving direct projections from Mrgprb4-lineage neurons.^179^^,^^181^ These spinoparabrachial Gpr83+ neurons contribute to processing of affective somatosensorial features, and depending on stimulus intensity, their activation can be involved in positive and negative valence.^181^ Prokineticin receptor 2-expressing sensory interneurons (PROKR2) via their interaction with their ligand, PROK2, were shown to respond to gentle stroking and linked to rewarding stimuli in mice.^182^ Genetic ablation of PROKR2 spinal neurons abolished a pleasant touch-induced conditioned place preference, sparing itch and pain behavior in mice, suggesting their specificity in pleasant touch.^182^

Thus, these cell ensembles form a dedicated skin-to-brain circuit that is essential for social reward, sexual receptivity, and stress resilience, acting through activation of brain reward centers, such as the NAc and the VTA.^179^ Nevertheless, it remains to be determined to what extent the brain circuits discussed below—those involved in positive social interactions—also contribute to encoding the rewarding aspects of affiliative touch.

### Central integration of positive social interactions and affiliative touch

Following peripheral signal encoding of an external stimulus, touch cues are transmitted to second order neurons in the spinal cord, brainstem, and thalamus and finally, third order neurons carry the signal to the somatosensory cortex for further processing.^168^ The somatosensory cortex is involved in pain and emotional facial perception, as well as in prosocial behavior.^183^^,^^184^ Recent evidence from neural synchronization experiments in humans suggested that inter-brain synchrony in the range of alpha oscillations (7–13 Hz) in the somatosensory cortex can modulate social-touch induced analgesia.^185^ The barrel cortex, a specialized part of the primary somatosensory cortex in rodents, participates in social touch, showing distinct response characteristics upon social facial versus object touch stimuli.^164^^,^^186^

In humans, the mesolimbic dopamine pathway, including the NAc (or ventral striatum) and the VTA have been linked with processing social—but also non-social—rewards. Specifically, the striatum is a heterogeneous region, in terms of cell types, and its anatomy and high connectivity to other key brain regions place it in a prime position to integrate cognitive, affective and motor processes.^187^ The discovery of the reward prediction error (RPE; Box 1) with the seminal experiments of Wolfram Schultz with nonhuman primates, showing that midbrain dopamine neurons can respond to rewards or reward-associated cues,^188^ paved the way for examining reward-related responses in the striatal regions of human participants. BOLD (blood-oxygen-level-dependent) signals in fMRI experiments were reported to correlate with RPE-relevant signals especially during passive and instrumental learning.^189^^,^^190^^,^^191^^,^^192^ The RPE discovery has thus provided the framework for explaining behavioral adaptations based on past experiences.^193^ Activity of striatal regions in humans has been linked with social judgments and decisions, learning about others, cooperation and following social norms.^187^^,^^194^ Interestingly, people reported more excitement when sharing their monetary gains with a friend rather than a computer or stranger and this was also reflected in higher striatal BOLD activity.^195^ The VTA provides dopaminergic input to the striatum and has been implicated in the rewarding aspects of social stimuli.^196^ Prefrontal cortex regions are also participating in social reward processing, with the ventromedial prefrontal cortex (VMPFC) and orbitofrontal cortex shown to play a role in valuation of social rewards, decision-making in social contexts, and integrating emotional and social information, including processing of affective touch.^196^^,^^197^ Moreover, the mPFC has been shown to participate in self-referential processing, and moderating goal-directed social behaviors.^198^ The ACC seems to be engaged during the anticipation and receipt of social rewards, and in monitoring social outcomes.^199^^,^^200^ It is important to note that many of the brain areas involved in pleasure, are also participating in pain processing, with data suggesting that these two processes can be modulated by distinct neuronal populations within overlapping regions.^181^^,^^201^

The DMN, an organization of many cortical regions, such as parietal, temporal, and prefrontal areas is shown to be involved in social cognition^202^^,^^203^ and has recently been linked with social closeness.^204^ Moreover, the anterior insula, among its many functions, appears to be important in encoding social familiarity or novelty with fMRI studies indicating its involvement in affective and evaluative components of empathy.^205^^,^^206^ Finally, the amygdala and the hippocampus have been shown to process several aspects of social interactions, including emotional and mnemonic encoding of rewarding social experiences, respectively.^207^^,^^208^^,^^209^^,^^210^^,^^211^

In mice, prosocial contact involving tactile cues seems to be a rewarding experience as they actively work to obtain access to interactions with an unfamiliar conspecific, with VTA DA neurons encoding social prediction error and driving social reinforcement learning.^212^ Moreover, rodents prefer social touch-associated chambers in conditioned place preference tasks.^182^ Please note, however, that in autism mouse models such as the *Fmr1* KO mice, it was shown that there was increased avoidance and aversive facial expressions to social, rather than to object touch.^19^^,^^20^ Hypoactivation of brain areas involved in the processing of the social component of touch, in parallel to enhanced reactivity to non-social touch processing, as well as pain hypersensitivity, have also been reported in individuals diagnosed with autism. This could possibly account for their aversion toward touch approaches and subsequent social withdrawal.^213^^,^^214^^,^^215^^,^^216^

Similar to humans, mesolimbic areas such as the VTA and the NAc are involved in social processing of incentives, reward from social interactions and reinforcing rewarding social interactions in rodents.^198^^,^^207^ Interestingly, manipulation of these areas by downregulating autism-related genes early in life has been shown to induce sociability deficits highlighting their crucial involvement in social behavior.^217^^,^^218^ Thus, the mouse VTA is shown to have a central position in mediating valence attribution, including the rewarding components of positive social interactions.^212^ Areas of the prefrontal cortex, such as the mPFC, prelimbic cortex (PL), infralimbic cortex (IL) and anterior cingulate cortex (ACC) are central to social cognition, decision-making, and regulating social behaviors and social emotional processing.^89^^,^^219^ The amygdala and hippocampus are also involved in emotional, motivational, and mnemonic aspects of social behavior.^198^^,^^207^ Interestingly, using a head-fixed assay, different aspects of social touch could be assessed along with accompanying changes in brain region activation.^19^ The authors showed that touch contexts (social vs. object) could be decoded by population activity in the tail of the striatum (tSTR), vibrissal somatosensory cortex (vS1), and the basolateral amygdala.^19^

Another hub of subregions involved in a plethora of processes including stress responses, social attachment, and affiliative behaviors is the hypothalamus. For example, oxytocin parvocellular neurons of the paraventricular nucleus (PVN) of the hypothalamus are shown to be involved in social touch behaviors between female rats.^220^ Finally, as in humans, the anterior part of the insular cortex in mice is shown to be involved in social behaviors such as recognition memory.^221^

## Positive effects of social touch

We have already mentioned the importance of social ties in general health, with reduced integration in social contexts being linked with increased risk of mortality and with social isolation resulting in many problematic outcomes.^5^^,^^6^ But zooming in, how and in what way are touch interventions beneficial? Further we review select examples from the literature and summarize key findings from recent systematic reviews.

Social touch between romantic partners while one of them was receiving experimental pain stimuli was shown to induce pain reduction and this effect was hypothesized to be due to empathy between couples.^222^ Another study showed that touch coming from romantic partners could reduce feelings of jealousy (induced during the experiment) in anxiously attached individuals and this effect was not observed in control conditions including non-touch interventions.^223^ Interestingly, salivary cortisol, alpha amylase, and emotional responses to specialized questionnaires, denoted that a hand-massage can have beneficial effects for participants, including the highly self-critical individuals.^224^ Moreover, physical contact provided by partners prior to a social stress was associated with lower cortisol and decreased heart rate in women.^225^ In mice it was demonstrated that unstressed partners increase allogrooming behavior toward stressed partners and this results in alleviation of anxiety-like behavior.^160^ Similar consolation behaviors toward stressed individuals have also been observed in voles.^159^^,^^226^

In a systematic review and meta-analysis, it was documented that indeed touch interventions have significant beneficial effects for mental health components in adults and newborns.^227^ The effect sizes in this study were reported as medium but notably, touch was most effective for reducing pain, depression, and anxiety in adults and children, and for promoting weight gain in newborns. Many mechanisms have been proposed for the mechanistic effects of touch’s beneficial effects, including relational-cognitive and neurobiological changes.^228^

It is important to note that indications of altered social touch processing have been described to manifest along with symptoms of hypervigilance, anhedonia, and negative emotions in a subset of individuals affected by traumatic social experiences. In fact, stress exposure can exert effects on social touch processing and sensory integration affecting its rewarding properties.^22^ Affected domains include altered activation of the primary somatosensory cortex upon suboptimal activation of C-tactile fibers, aberrant insular activity and connectivity and, perturbed peptide signaling, such as oxytocin and ghrelin.^22^ Additional lines of evidence suggest reduced hippocampal and enhanced superior temporal gyrus (STG) activation during aversively-perceived social touch in a subset of PTSD patients who reported the experience of traumatic events in the past.^229^ In other cases, however, traumatic experiences fail to alter the perception of social touch, highlighting the increased variability in stress responses.^230^

Nevertheless, taken together, evidence from the literature so far has demonstrated that social touch can improve several aspects in the life of humans and rodents (and of course of other social species), including pain alleviation, stress hormone reduction, mood and anxiety behavior improvements, amelioration of the immune system, and developmental benefits. Indeed, social touch appears to mitigate the effects of social stress across diverse physiological domains (Figure 2). For instance, oxytocin mediates positive social interactions within in-groups, such as between mother-infant and intimate partners, whereas oxytocin signaling seems to be dysregulated following exposure to stress.^231^^,^^232^^,^^233^^,^^234^ Both preclinical and clinical data suggest that oxytocin release upon social touch could underlie its anxiolytic and beneficial effects in counteracting stress effects, which mostly manifest as reduced corticosterone responses, restored social interactions and, in the case of humans, decreased systolic blood pressure, and diminished subjective experience of social distress.^235^^,^^236^^,^^237^^,^^238^^,^^239^^,^^240^^,^^241^^,^^242^

Despite the emerging evidence (summarized in Packheiser et al., 2024^227^), touch interventions or somatosensory approaches are not yet a standard treatment regimen in trauma- and stress-related disorders. Rather these interventions are considered as adjunctive or supplementary to CBT, EMDR, and medications. Building a strong theoretical background by orienting basic and clinical research toward uncovering the mechanisms via which social touch drives social buffering could eventually determine whether these avenues could be considered first line treatments for trauma- and stress-related disorders. Further we discuss some of the specific open questions associated with social stress and social touch.

## Unresolved questions pertaining to social stress

Advancements in contextual fear studies have provided valuable information on the neurobiology of trauma- and stressor-related disorders,^47^ yet research is now uncovering the unique nature of social stress in eliciting distinct neurobiological signatures.^90^ Thus, the degree to which social stress responses differ from those of other stressors, such as footshocks, in terms of engaged circuitries and affected mechanisms remains elusive. Direct comparisons are necessary; however, several technical and methodological limitations regarding the quantification of elicited aggression, as compared to the more controllable nature of footshocks, emerge and need to be taken into consideration. Toward this direction and given the dual role of the social stress-responding regions like the VTA and LHb in both reward and aversion, more attention should be given on how such regions project to other brain areas that are traditionally thought to participate in contextual or auditory fear processing.^45^^,^^110^^,^^243^^,^^244^ Moreover, in light of the diverse roles attributed to many of the brain regions involved in social defeat, it is likely that cell-specific adaptations within the same region, such as MSN neurons in the NAc, could be driving further specialization and distinct responses and consequently, call for deeper analysis.

Going one step further, our current understanding of the way that social stress affects memory processes, ultimately leading to overgeneralization and fear relapse, remains incomplete.^245^^,^^246^ The vast majority of basic research has mainly focused on the acute effects of social stress while less is known about its long-term implications. Nevertheless, characterizing the long-term consequences of social stress is one of the most relevant questions with translational significance. Moreover, greater emphasis should be placed on how chronic social stress influences developmental trajectories in critical periods, like childhood and adolescence, as well as during adulthood and aging. The majority of results and protocols discussed in the present review focus on social stress during adulthood; however, infancy, childhood, and adolescence constitute sensitive periods where major processes and systems are under development.^247^^,^^248^ Consequently, exposure to adversities such as neglect, domestic violence or poverty can pose long-lasting impacts on brain physiology and promote vulnerability to subsequent challenges later in life.^249^^,^^250^ For instance, early life stress—in the form of maternal separation or limited bedding and nesting material—has been shown to affect a range of domains, such as subsequent stress transfer, corticosterone reactivity, dynamics of circuits involved in sociability, and gene transcription in key stress-responsive regions engaged during subordination.^251^^,^^252^^,^^253^ Recent studies demonstrate the detrimental effects of challenges, such as postweaning social isolation, for cortical development, microglial integrity, and social behavior.^165^^,^^254^ The peripubertal period has been established as a particularly sensitive period for stress programming effects with peripubertally stressed rats showing heightened anxiety-like behavior, aggression and deficits in cognitive processes in adulthood.^255^^,^^256^^,^^257^^,^^258^ It should be noted that certain effects of early life stress have been reported to be transferrable *trans*-generationally in multiple animal models.^10^^,^^259^^,^^260^

In parallel, mounting evidence is starting to uncover systemic effects of social defeat that go beyond the brain, with targets including the heart,^261^ liver,^262^ gut,^263^ spleen, and lung.^264^ Therefore, elucidating such inter-organ communication readouts in social stress susceptibility holds significant translational potential and urges multi-disciplinary research and collaboration. Hence, prioritizing individual differences in social stress research will set the groundwork for identifying robust behavioral and physiological biomarkers for susceptibility, potentially expanding the efficacy of clinical interventions.

According to the 2018 global estimates, 30% of women aged 15 years and older have experienced intimate partner violence or non-partner sexual violence, with the true prevalence of the latter being speculated to reach even higher levels in low- and middle-income countries due to fear of exposure.^265^ The extent to which social stress affects behavioral modalities, brain regions, and mechanisms similarly (or not) across sexes has yet to be determined. Improving the precision of our behavioral paradigms and developing sensitive, female-tailored readouts to understand female responses will shed light on sex differences underlying stress-related disorders.

## Unresolved questions pertaining to social touch

Despite the acknowledgment of the beneficial effects of social touch, there are still significant gaps in our knowledge. The research regarding social touch in humans has been documented to include a large variability in terms of studied population (e.g., adults, children, and newborns), the type of social touch (e.g., a hug or a massage), the health readouts assessed post-intervention and the agent of social touch (e.g., partner vs. professional or stranger).^227^ This increased variability in human studies, combined with the lack of animal studies that examine this topic are slowing progress on the mechanistic aspects regarding the beneficial effects of social touch.

Although it is shown that increasing social tactile experiences or affiliative touch behaviors in several species, including humans and mice,^160^^,^^266^ can reduce negative stress effects acutely, whether these interventions would have the potential to regulate social stress effects in the long-term, has not been fully addressed. This is a critical topic as depending on the answer, the policies of stress management could be steered toward systematically incorporating such practices.

Thus, despite somatosensory approaches being available and seemingly beneficial for the treatment of trauma- and stress-related disorders, such as PTSD, more research is needed to form a robust theoretical background for establishing this type of approach as a standard treatment option.^267^ The brain mechanisms that are involved in stress-alleviating effects of social touch are not thoroughly mapped yet.^160^ For example, whether touch-related plasticity in the brain would potentially involve structural or functional changes and the relationship between them, has not been thoroughly addressed.^268^ Whether neurobiological signatures, such as RPE signals, observed in the context of other rewarding experiences, are also involved during affiliative touch remains to be explicitly investigated. Moreover, how peripheral signal processing, starting with touch neurons on the skin, is propagated in the brain to induce these potential RPE signals and how these processes are then integrated to buffer stress effects needs to be systematically dissected. Although there is substantial evidence on the physiology of peripheral detection of touch, identifying the specific mechanisms by which social stress can potentially influence tactile sensory processing remains a key research priority. It must be noted that despite the findings reviewed here regarding brain regions and cells bridging the periphery-to-brain interactions, other regions and cell types, including non-neuronal subtypes, will very likely be uncovered in the future to play important roles in social behaviors and touch. Therefore, interdisciplinary approaches prioritizing well-thought behavioral paradigms and machine learning-based analyses,^269^^,^^270^ exploratory cell activity mapping of brain-wide datasets,^271^ incorporating other markers capturing inhibition rather than excitation,^272^ as well as, imperatively including females in our animal research,^273^ are necessary steps to expand our understanding in an impactful manner.

Thus, while further research is clearly needed, it is important to highlight that the existence of these parallel somatosensory systems in humans and other species underscores the evolutionary conservation of touch as a core mechanism regulating social behavior and emotional well-being.^274^^,^^275^ This evolutionary continuity provides a strong foundation for important translational research.

## Conclusions and synthesis

Our previous discussion reveals that the neurobiological mechanisms pertaining to both social stress and affiliative touch concern many shared cortical and subcortical regions (including limbic areas and the midbrain). By reviewing the literature from this angle, it also becomes evident that equivalent brain areas are implicated for both affective states in humans and rodents. Thus, we conclude that basic research investigations including cell specific- (not exclusively focused on neurons) and projection-specific dissection are required to drive the field forward.

Sources of social stress in the form of bullying, war, conflict, and intimate partner violence, are continuously relevant and present in our society. Prevalence of anxiety and stress-related disorders is high, whereas effective treatments are still lacking.^47^^,^^50^^,^^276^ As basic scientists, we are deeply motivated by the hope that our research will lead to testable hypotheses and answers that will improve disease symptomatology and patient quality of life. Here, we review evidence that demonstrates that whereas it is known that prosocial experiences can have an overall positive effect on well-being, the potential of a positive social experience, such as affiliative touch, in ameliorating long-term consequences of stress, has not been systematically studied and thus, it remains largely unexploited as a standard treatment avenue. Although similar ideas were mentioned previously in terms of prosocial training,^277^ the actual clinical and psychotherapeutic practice has not significantly changed.

Beyond distinct physiological adaptations underlying stress alleviation, social touch has been implicated in a broader state of negative affect regulation, promoting social allostasis and (meta) cognition.^278^ Recently, a two-brain model of comforting touch was proposed to explain the positive effects of social touch on physical pain and emotional distress alleviation. More specifically, the authors described a feedback loop between the toucher and the receiver, where distinct processes, namely brain-to-brain coupling, activation of reward circuits, and emotional regulation processing, take place and orchestrate this loop.^279^ Admittedly, however, a large volume of research so far has focused on collecting data from individuals as they were single entities whereas many authors have emphasized the need to address interacting individuals in tandem by explicitly assessing inter-brain synchronization.^279^^,^^280^^,^^281^ Potentially, incorporating research that considers second-person neuroscience perspectives could significantly enhance our understanding of both the shared and distinct neural circuits involved in pain and emotional stress and how these experiences can be modulated by social touch.

It is important to note that the type of stressor and the developmental period when it is encountered can largely interact with the genetic and behavioral makeup of the individual, thus affecting the degree to which social touch can be perceived as beneficial.^22^ For instance, severe childhood maltreatment was documented to affect several domains of social touch processing, leading to discomfort associated with specific to touch patterns and to a preference for larger interpersonal distances.^282^^,^^283^ Moreover, as mentioned previously, individuals diagnosed with autism can be particularly sensitive to stressful events and due to difficulties engaging in social interactions, they often lack resources to buffer trauma and stress effects via communication and social support.^284^^,^^285^ Future research will need to identify interventions that can potentially reverse or, at least, ameliorate negative perceptions of social affectionate touch in these cases. For example, dissecting which exact features of touch contribute to its unpleasantness in specific populations (sensory, psychological, attachment, social, or cultural factors) can aid in devising alternative strategies that could still offer benefits to patients.

Regarding affiliative social behaviors, social touch evoking positive affect seems to be particularly promising in alleviating stress effects, at least acutely. Basic research using rodents or other social species as model organisms should validate the role of touch in ameliorating the negative consequences of social stress in the long-term. Based on our perspective here, where social stress can be perceived by the individual as a “breach” of social trust and positive interactions, it would be intriguing to assess the specificity of such interventions to relieve symptoms after stress. Namely, it should be systematically tested whether this particular type of prosocial behavior, rather than other interventions, can be more efficient to relieve social stress effects.

The general idea that experiencing positive emotions could be the basis for ameliorating negative ones generated by previous traumatic experiences is not novel. A similar concept from the field of psychotherapy was proposed in the 40s by Alexander and French and termed as corrective emotional experience to describe the change that can occur to painful emotional processing in the context of patient-therapist relationships.^286^^,^^287^ However, here, our view is to extend this concept beyond the temporal and spatial confines of an actual therapeutic session. From the neuroscience field, it is well-established that therapeutic changes are associated with emotional arousal and memory reconsolidation-mediated emotional updating.^288^ We propose that focusing on expanding core knowledge of how prosocial rewarding somatosensorial experiences, such as social touch, can offer both immediate and long-term beneficial effects in stress alleviation, can be a determining factor for shaping future treatment strategies regarding stress-related disorders.

Therefore, while the positive impact of social buffering, such as affiliative touch on stress relief, pain reduction, health outcomes, and even mortality is well established, we believe that these valuable insights have yet to be fully harnessed to shape effective policies and treatment approaches. Along these lines, McEwen and Davidson have suggested that incorporating training in areas such as kindness, meditation, and mindfulness could be beneficial.^268^ Moreover, other important parameters such as the agent of buffering, i.e., whether these interventions are performed from family members vs. strangers should be taken into account for future research.^6^ Regarding individuals with severe distress to social touch, alternatives such as intranasal oxytocin administration could be considered.^289^ Here, we propose that social buffering via affiliative touch approaches could potentially be one of the most important buffering factors for social stress, potentially counteracting detrimental social stress effects (for a proposed model and examples please see Figure 2). We believe that this concept can serve as the foundation for various testable hypotheses. For instance, one could investigate in animal models whether individual differences in openness to social interactions, such as permitting allogrooming behaviors from conspecifics, would be associated with greater resilience in the face of social stress.

Exploring the physiological mechanisms of these precise social buffering effects and understanding the associated plasticity and structural/functional changes, will require interdisciplinary collaboration and communication across scientific domains. Until recently, discoveries regarding this topic remained isolated within specific fields.^153^ However, by default, studying positive experiences, such as affiliative social touch, encompasses multiple factors, including the evolution of each species and spans from the individual to society as a whole. Therefore, it is plausible that increasing the dialogue between fields, such as psychology, neuroscience, sociology, and evolutionary biology will yield original and translationally relevant research projects. Although this is a challenging undertaking, we believe that, in the long run, this interdisciplinary effort can play a crucial role in advancing the treatment of stress- and anxiety-related disorders. If indeed, augmenting prosocial behaviors is proven to be beneficial in the long-term for the brain and behavior in the aftermath of social stress, this line of research can potentially guide policy making by integrating non-invasive, low-cost programs to enhance positive social interactions.

## Acknowledgments

We sincerely thank Dr. Christina Seryianni for her valuable input and constructive critique, informed by her professional perspective as a psychotherapist/psychologist.

Figures and schematics were designed in Biorender.com.

The laboratory of ST receives financial support from the 10.13039/501100001711Swiss National Science Foundation (SNSF), the 10.13039/501100022761Pierre Mercier Foundation, and the 10.13039/501100006390University of Lausanne (UNIL) Sophie Afenduli Foundation (accredited to L.P.P.).

## Author contributions

All authors performed the literature search and drafted the initial manuscript. L.P.P. contributed to the organization and interpretation of the content. S.T. conceptualized the topic and content of the review. All authors read and approved the manuscript in its final version.

## Declaration of interests

The authors declare no competing interests.
